# Enterotoxin Production of *Bacillus thuringiensis* Isolates From Biopesticides, Foods, and Outbreaks

**DOI:** 10.3389/fmicb.2018.01915

**Published:** 2018-08-23

**Authors:** Sophia Johler, Eva M. Kalbhenn, Nicole Heini, Peter Brodmann, Sylvia Gautsch, Murat Bağcioğlu, Matthias Contzen, Roger Stephan, Monika Ehling-Schulz

**Affiliations:** ^1^Institute for Food Safety and Hygiene, University of Zurich, Zurich, Switzerland; ^2^Functional Microbiology, Institute of Microbiology, Department of Pathobiology, University of Veterinary Medicine Vienna, Vienna, Austria; ^3^Kantonales Labor Basel-Stadt, Basel, Switzerland; ^4^Chemisches und Veterinäruntersuchungsamt Stuttgart, Fellbach, Germany

**Keywords:** *Bacillus thuringiensis*, *Bacillus cereus* group, enterotoxicity, Vero cell assay, sphingomyelinase

## Abstract

While the relevance of *Bacillus (B.) cereus* as a major cause of gastroenteritis is undisputed, the role of the closely related *B. thuringiensis* in foodborne disease is unclear. *B. thuringiensis* strains frequently harbor enterotoxin genes. However, the organism has only very rarely been associated with foodborne outbreaks, possibly due to the fact that during outbreak investigations, *B. cereus* is routinely not differentiated from *B. thuringiensis*. A recent EFSA scientific opinion stresses the urgent need for further data allowing for improved risk assessment, in particular as *B. thuringiensis* is a commonly used biopesticide. Therefore, the aim of this study was to gain further insights into the hazardous potential of *B. thuringiensis*. To this end, 39 *B. thuringiensis* isolates obtained from commercially used biopesticides, various food sources, as well as from foodborne outbreaks were characterized by *panC* typing, *panC*-based SplitsTree analysis, toxin gene profiling, FTIR spectroscopic analysis, a cytotoxicity assay screening for enterotoxic activity, and a sphingomyelinase assay. The majority of the tested *B. thuringiensis* isolates exhibited low (23%, *n* = 9) or mid level enterotoxicity (74%, *n* = 29), and produced either no (59%, *n* = 23) or low levels (33%, *n* = 13) of sphingomyelinase, which is reported to act synergistically with enterotoxins Nhe and Hbl. One strain isolated from rosemary was however classified as highly enterotoxic surpassing the cytotoxic activity of the high-level reference strain by a factor of 1.5. This strain also produced vast amounts of sphingomyelinase. Combining all results obtained in this study into a fingerprint pattern, several enterotoxic biopesticide strains were indistinguishable from those of isolates from foods or collected in association with outbreaks. Our study shows that many *B. thuringiensis* biopesticide strains exhibit mid-level cytotoxicity in a Vero cell assay and that some of these strains cannot be differentiated from isolates collected from foods or in association with outbreaks. Thus, we demonstrate that the use of *B. thuringiensis* strains as biopesticides can represent a food safety risk, underpinning the importance of assessing the hazardous potential of each strain and formulation used.

## Introduction

The *Bacillus cereus sensu lato* group comprises *B. cereus sensu strictu* and multiple other closely related species, including *B. thuringiensis* (Schnepf et al., 1998; Ehling-Schulz et al., 2011). *B. thuringiensis* and *B. cereus s. s*. are genetically intermingled and can only be differentiated by the presence or absence of insecticidal toxins, which are delineating the species *B. thuringiensis* (Schnepf et al., 1998; Ehling-Schulz et al., 2011). Since these two species are indistinguishable using cultural detection methods or 16S rDNA sequencing (Ehling-Schulz and Messelhäusser, 2013), they have been suggested to represent the same species (Helgason et al., 2000).

*B. cereus s. s*. causes two distinct forms of gastrointestinal disease—the diarrheal and the emetic type of *B. cereus* gastroenteritis. The emetic syndrome is caused by oral intake of the emetic toxin cereulide, which elicits nausea and vomiting and is mostly associated with cooked rice dishes (McKillip, 2000; Ehling-Schulz et al., 2004). By contrast, the diarrheal syndrome is caused by the heat-labile enterotoxins Nhe, Hbl, and CytK (Stenfors Arnesen et al., 2008) and is often associated with contaminated meats, sauces, and dairy products (McKillip, 2000). Sphingomyelinase (SMase), a virulence factor structurally related to *Staphylococcus aureus* beta toxin, has been reported to interact synergistically with Nhe and Hbl. SMase has been shown to enhance Hbl hemolysis (Beecher and Wong, 2000) as well as *in vitro* cytotoxicity (Doll et al., 2013). Furthermore, strains producing high levels of SMase that had been isolated from patients with sepsis and endophthalmitis were found to be lethal in mice (Oda et al., 2012), suggesting that the contribution of SMase to *B. cereus* virulence may have been underestimated. Results from *in vivo* studies using *B. cereus* mutants foster the hypothesis that SMase also enhances enterotoxin-mediated cytotoxicity in the human host (Oda et al., 2010; Doll et al., 2013).

Since *B. thuringiensis* has been reported to produce enterotoxins (Griffiths, 1990; Damgaard et al., 1996; Gaviria Rivera et al., 2000) and is routinely not differentiated from *B. cereus s.s*. by diagnostic laboratories, some outbreaks of food poisoning attributed to *B. cereus* may in fact have been caused by *B. thuringiensis*. This is of particular interest, since *B. thuringiensis* is widely used as biopesticide in organic farming on account of the pronounced insecticidal effects of crystal proteins (Cry toxins), which are formed during sporulation of *B. thuringiensis* (Bravo et al., 2011). Most biopesticide formulations used contain both insecticidal proteins and spores (EFSA BIOHAZ Panel, 2016).

Previous studies have demonstrated *B. thuringiensis* enterotoxin expression using immunological assays (Damgaard, 1995; Hansen and Hendriksen, 2001; Yang et al., 2003). However, these assays only recognize certain enterotoxin subunits (EFSA BIOHAZ Panel, 2016) and are not a suitable predictor of cytotoxicity (Miller et al., 2018). Data on *B. thuringiensis* enterotoxin production generated using bioassays are scarce. Damgaard et al. (1996) and Gaviria Rivera et al. (2000) were able to show that culture supernatants of *B. thuringiensis* isolates inhibited [^14^C]-leucine uptake in a Vero cell assay. However, the authors only included three biopesticide strains HD-1 (serotype *kurstaki*), HD-567 (serotype *israelensis*), and NB-125 (serotype *tenebrionis*). In particular, ABTS-1857 (serotype *aizawai*), which has been discussed as causative agent of a foodborne outbreak in Germany in 2012 (EFSA BIOHAZ Panel, 2016), was missing.

In spite of these findings, the relevance of *B. thuringiensis* as causative agent of foodborne disease is controversially discussed (Jackson et al., 1995; McIntyre et al., 2008; EFSA BIOHAZ Panel, 2016; Raymond and Federici, 2017) and the EFSA recently published a scientific opinion (EFSA BIOHAZ Panel, 2016) stressing the urgent need for further data in order to improve risk assessment.

Therefore, in this study, we chose a systematic approach to gain further insights into the hazardous potential of naturally occurring and commercially used *B. thuringiensis*. To that end, a collection of *B. thuringiensis* isolates obtained from commercially used biopesticides, foods, and outbreaks was characterized using *panC* typing, *panC-*based SplitsTree analysis, toxin gene profiling, FTIR-spectroscopic analysis, a cytotoxicity assay, and a SMase assay.

## Materials and methods

### Bacterial strains

This study includes a total of 39 *B. thuringiensis* isolates. Eight isolates from biopesticides (Table 1), 24 isolates from foods (Table 2), and seven isolates linked to three foodborne outbreaks (Table 3). *B. cereus sensu lato* were isolated from foods following standard routine procedures by plating samples either directly or after homogenization in serial dilutions on selective media (MYP) and with incubation at 30 and 37°C, respectively. For strains associated with outbreak investigations, additional information on the isolation context is provided elsewhere (EFSA BIOHAZ Panel, 2016; Schmid et al., 2016). Isolates from biopesticides were obtained by plating of serial dilutions of the respective biopesticides on MYP agar.

**Table 1:** Background information on the seven *Bacillus thuringiensis* biopesticide strains included in this study.

**Biopesticide strain^a^**	***B. thuringiensis* subspecies**	**Isolate ID in this study**
GC-91	*aizawai*	CH_186
ABTS-1857	*aizawai*	CH_181
		CH_185
B401	*aizawai*	P05_2
SA-11	*kurstaki*	CH_164
ABTS-351	*kurstaki*	CH_183
Solbac	*israelensis*	CH_133
NB-176	*morrisoni* (var. *tenebrionis*)	CH_187

a *If no strain was specified on the product, trade names are given*.

**Table 2:** Background information on the *Bacillus thuringiensis* isolates collected from food sources included in this study.

**Strain ID**	**Food source**	**Isolation context**
CH_9	Heated chicken breast	Army catering facility
CH_10	Heated tomatoes	Army catering facility
CH_19	Pork roast	Army catering facility
CH_24	Heated potatoes	Army catering facility
CH_26	Pollack filet & sauce	Army catering facility
CH_34	Runner beans	Army catering facility
CH_35	Ratatouille	Army catering facility
CH_40	Rosemary	Retail level
CH_41	Asia Mix (peppermint, coriander, thai chives)	Retail level
CH_42	Organic oregano	Retail level
CH_43	Organic sage	Retail level
CH_44	Organic peppermint	Retail level
CH_48	Rosemary	Retail level
CH_50	Organic coriander	Retail level
CH_65	Tarragon	Retail level
CH_66	Basil	Retail level
CH_69	Lasagna (precooked)	Surveillance
CH_72	Vegetable juice (spinach, carrot, cucumber, mint)	Surveillance
CH_81	Sauce (precooked)	Surveillance
CH_95	Sushi	Retail level
CH_96	Sushi	Retail level
CH_160	Heated pasta	Surveillance
P01_1	Honey	Self-surveillance
P01_3	Honey	Self-surveillance

**Table 3:** Background information on the seven *Bacillus thuringiensis* isolates collected in association with outbreaks.

**Outbreak**	**Strain ID**	**Sample**	**Source^a^**	**Year of isolation**
Lower Austria	2/27/S	Human feces	Vetmeduni	2013
Lower Austria	6/27/S	Human feces	Vetmeduni	2013
Lower Austria	1/29 AGES	Fruit salad	AGES	2013
Linz	3/22 AGES	Bell pepper	AGES	2013
Germany	CVUAS 2492	Lettuce	CVUAS	2012
Germany	CVUAS 9660	Lettuce	CVUAS	2012
Germany	CVUAS 9659	Lettuce	CVUAS	2012

a *AGES, Austrian Agency for Health and Food Safety; CVUAS, Chemisches und Veterinäruntersuchungsamt Stuttgart; Vetmeduni, University of Veterinary MedicineVienna*.

Screening for parasporal crystal bodies enabled *B. thuringiensis* species identification and was performed as follows: Isolates were grown on T3 agar (Travers et al., 1987) for 3 days at 30°C. Sporulated culture material was resuspended in 10 μL sterile deionized water and immediately screened for parasporal crystals of diamond, bipyramidal, or spherical shape using phase contrast microscopy and oil immersion (EFSA BIOHAZ Panel, 2016).

### *panC* typing and *panC*-based SplitsTree analysis

All strains included in the study were subjected to *panC* typing for assignment to phylogenetic groups using the pantothenate synthetase gene as previously described (Guinebretière et al., 2008). Cluster analysis of *panC* nucleotide sequences was performed using the SplitsTree™ software (http://www.splitstree.org). Several reference strains were included in the SplitsTree analysis (panC type I: DSM 12442; panC type II: WSBC10311; panC type III: Ames; panC type IV: ATCC 14579; panC type V: BCT-7112; panC type VI: WSBC 10204; panC type VII: NVH391-98).

### FTIR spectroscopic fingerprinting

The strains were grown as lawns on tryptone soy agar (TSA) plates (Oxoid, Wesel, Germany) at 25°C for 24 h ± 30 min. Samples were prepared as described by previously (Oberreuter et al., 2002; Ehling-Schulz et al., 2005). Briefly, one loop of cell material was suspended in 100 μL sterile deionized water. Isolates yielding a clumpy suspension were subjected to ultrasonication for 5 × 1 s at 100% power with a Bandelin Sonopuls HD2200 (Bandelin electronic, Berlin) in order to improve spectral quality. Bacterial suspension were spotted on a zinc selenite (ZnSe) optical plate and dried at 40°C for 30 min. Infrared absorption spectra were recorded, using a HTS-XT microplate adapter coupled to a Tensor 27 FTIR spectrometer (Bruker Optics GmbH, Ettlingen, Germany). Spectral acquisition was performed in transmission mode in the spectral range of 4,000–500 cm^−1^ using the following parameters: 6 cm^−1^ spectral resolution, zero-filling factor 4, Blackmann-Harris 3-term apodization, and 32 interferogramms were averaged with background subtraction for each spectrum. Independent measurements were prepared per strain to yield the number of spectra per strain required for cluster analysis. The quality of FTIR spectral data was evaluated first using OPUS software (version 7.5; Bruker Optics, Ettlingen, Germany). Additionally, second derivatives were calculated using the Savitzky-Golay algorithm with 11 smoothing points and the derivative spectra were unit vector normalized subsequently for further data processing. The spectral region of 1,500–800 cm^−1^ was chosen as fingerprint region and FTIR spectral data were subjected to hierarchical cluster analysis (HCA) using the Ward's algorithm. Further, the FTIR data set was tested against the normality of the data by using Mardia and Royston tests (Mecklin and Mundform, 2004). These resulted in a non-normality assumption (the degree of significance, *p* < 0.0001), which is considered as monotonic but also non-linear distribution. Thus, spearman rank correlation was utilized for analysis of this FTIR data set, showing non-parametric statistic monotonic association between the variables. Spectral pre-processing and multivariate data analysis of FTIR spectra were performed using the Unscrambler X (version 10.5, Camo AS, Norway) and Orange data mining toolbox for Python (software version 3.13.0; Demšar et al., 2013).

### Toxin gene profiling

All isolates were screened for the presence of toxin genes *nheAB, hblDA, cytK*, and *ces*, coding for the non-hemolytic enterotoxin (Nhe), hemolysin BL (Hbl), cytotoxin K (CytK), and cereulide (Ces), respectively. PCR-based screening for toxin genes, as well as subsequent assignment to toxin profiles A–G was performed as previously described (Ehling-Schulz et al., 2006).

The following strains were used as positive controls: F1942/85 for *nhe, hbl, cytK* (isolated from an outbreak of *B. cereus* diarrheal disease) and F4810/72 for *ces* (isolated from vomit in a clinical case of *B. cereus* emetic disease). Details of strains used as control are provided elsewhere (Ehling-Schulz et al., 2005).

### Vero cell cytotoxicity assay

Cytotoxicity in a Vero cell assay was used to determine enterotoxin production. An overnight culture of each isolate in 3 mL CGY (16–18 h, 30°C, 120 rpm) was used to adjust a 30 mL CGY day culture in an Erlenmeyer to an OD_600_ of 0.05. The day cultures were incubated at 30°C (120 rpm shaking) until an OD_600_ of 7 was reached. A volume of 5 mL of each day culture was centrifuged at 11,000 rpm for 10 min to harvest the supernatant. Sterile filtrated supernatant aliquots of 1 mL were supplemented with 10 μL 0.1 M EDTA-Na_2_ and stored at −80°C. Cytotoxicity was subsequently determined using Vero cells as previously described (Moravek et al., 2006). The reciprocal titer of the reference strain NVH 0075-95, a *B. cereus* strain isolated from vegetable stew in a clinical case of *B. cereus* diarrheal disease in Norway in 1995 (Lund and Granum, 1996), was used for normalization of absolute values. Strains were classified as low level, mid level, or high level enterotoxin producers based on the classification described by Jeßberger et al. (2015), with cutoffs normalized based on the mean of the high level reference strain NVH 0075-95.

### Sphingomyelinase assay

Overnight cultures of all isolates (3 mL LB broth, 37°C, 120 rpm) were used to adjust 50 mL of LB broth to an OD_600_ of 0.05. All day cultures were subsequently grown to an OD_600_ of 4 at 37°C and 120 rpm shaking and harvested by centrifugation (6,500 × *g*, 4 min at 4°C). Cell pellets were discarded and supernatants were subjected to sterile filtration (pore size 0.2 μm). Six milliliters of sterile supernatant were concentrated to 100 μL using Vivaspin™ protein concentrator spin columns (GE Healthcare) with a cut-off size of 30,000 kDa. The sphingomyelinase (SMase) activity of the isolates was subsequently determined using the Amplex Red Sphingomyelinase Assay Kit (Invitrogen/Molecular Probes) in accordance with the manufacturer's instructions with minor changes: all reactions were developed for 20 min at 37°C in a light-protected 96-well microplate (Corning Costar Assay Plate, Sigma Aldrich). In total, only 0.1 μg of protein of each sample was used. Fluorescence was measured with extinction/emission wavelengths of 530/585 nm (SpectraMax® M3 Microplate Reader, Molecular Devices). Experiments were done in duplicate. To account for differences in protein concentrations between strains, the enzyme activity was normalized to the protein concentration of the respective supernatant using a Bradford-based protein assay (Roti-Quant, Carl Roth GmbH), resulting in enzyme activity expressed in mU per mg of protein. *B. cereus* strain NVH 0075-95 associated with a clinical case of *B. cereus* diarrheal disease (Lund and Granum, 1996) was used as a reference.

## Results

### *panC* typing and *panC* based SplitsTree analysis

**Figure 1:**
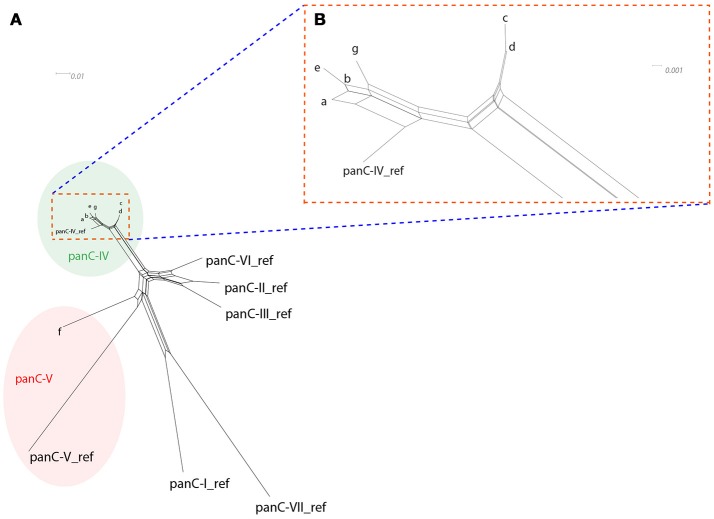
SplitsTree depicting the relatedness of the tested isolates based on *panC* sequence variations (**A**: overview over full SplitsTree, **(B)** detail zooming in on region depicting isolates assigned to *panC* type IV). SplitsTree generates unrooted phylogenetic networks from molecular sequence data. Proximity is used as an indicator of sequence similarity and thus relatedness of the respective isolates. Each of the 37 *B. thuringiensis* isolates exhibited one of only seven different *panC* nucleotide sequences. To improve readability of the network, these seven sequence variations are presented as clusters a-g. With the exception of one strain assigned to *panC* type V (CH_95, cluster f), all strains were assigned to *panC* type IV and clustered closely together, indicating that these isolates are closely related.

All strains included in the study were subjected to *panC* typing for assignment to phylogenetic groups using the pantothenate synthetase gene as previously described (Guinebretière et al., 2008). A comprehensive overview of *panC* types detected is presented in Table 4. With the exception of strain CH_95 assigned to *panC* type V, all other *B. thuringiensis* isolates were assigned to *panC* type IV. SplitsTree analysis was used to allow for higher resolution based on *panC* nucleotide sequences and clustered the tested isolates in seven groups designated a-g (see Figure 1). Cluster a comprised biopesticides strains ABTS-1857, GC-91, and B401, as well as the isolates obtained in association with the outbreak potentially caused by consumption of salad in Germany in 2012 and two human feces isolates from diarrheal disease patients during an outbreak in Austria. In addition, the cluster comprised four food isolates. Cluster b comprised biopesticide strains SA-11 and ABTS-351, as well as 15 food isolates and two outbreak associated isolates from Austria originating from food. Cluster c comprised the strain isolated from biopesticide Solbac, cluster d comprised NB-176 and one food isolate, and clusters e, f, and g exclusively comprised one food isolate each.

**Table 4:** Characterization results of the *Bacillus thuringiensis* isolates included in this study.

**Source^a^**	**Isolate ID**	**Toxin profile^b,c^**	**SplitsTree cluster**	**FTIR cluster^d^**	***panC typing***	**Enterotoxin production in the Vero assay**^**e**^			**SMase production**		
						**Normalized reciprocal titer**	**Std. Dev**.	**Classification**	**Normalized amplex red result**	**Std. Dev**.	**Classification**
P	CH_186	A	a	FTIR-A2	IV	0.3	0.0	Low	0.00	0.00	≤detection limit^f^
P	CH_181	A	a	FTIR-A1	IV	0.4	0.1	Mid	0.16	0.14	Low
P	CH_185	A	a	FTIR-A1	IV	0.5	0.2	Mid	0.06	0.05	Low
P	P05_2	A	a	FTIR-A1	IV	0.4	0.0	Mid	0.13	0.03	≤detection limit^f^
P	CH_164	A	b	FTIR-B2	IV	0.8	0.1	Mid	0.00	0.00	≤detection limit^f^
P	CH_183	A	b	FTIR-B2	IV	0.4	0.1	Mid	0.00	0.00	≤detection limit^f^
P	CH_133	A	c	FTIR-B1	IV	0.8	0.3	Mid	0.04	0.03	≤detection limit^f^
P	CH_187	C	d	FTIR-S	IV	0.2	0.0	Low	0.00	0.00	≤detection limit^f^
F	CH_9	D	b	FTIR-B2	IV	0.4	0.1	Mid	0.00	0.00	≤detection limit^f^
F	CH_10	D	b	FTIR-B2	IV	0.4	0.1	Mid	0.00	0.00	≤detection limit^f^
F	CH_19	D	b	FTIR-B2	IV	0.3	0.0	Low	0.00	0.00	≤detection limit^f^
F	CH_24	D	b	FTIR-B2	IV	0.4	0.1	Mid	0.00	0.00	≤detection limit^f^
F	CH_26	F	b	FTIR-A1	IV	0.5	0.1	Mid	0.00	0.00	≤detection limit^f^
F	CH_34	A	b	FTIR-B2	IV	0.2	0.0	Low	0.00	0.00	≤detection limit^f^
F	CH_35	A	b	FTIR-A1	IV	0.1	0.0	Low	0.00	0.00	≤detection limit^f^
F	CH_40	A	b	FTIR-B2	IV	0.8	0.1	Mid	0.00	0.00	≤detection limit^f^
F	CH_41	A	b	FTIR-A1	IV	0.8	0.1	Mid	0.00	0.00	≤detection limit^f^
F	CH_42	A	b	FTIR-B2	IV	0.7	0.0	Mid	0.00	0.00	≤detection limit^f^
F	CH_43	A	b	FTIR-A1	IV	0.8	0.1	Mid	0.00	0.00	≤detection limit^f^
F	CH_44	A	b	FTIR-B2	IV	0.6	0.1	Mid	0.00	0.00	≤detection limit^f^
F	CH_48	C	d	FTIR-B2	IV	1.5	0.3	High	1.21	0.12	High
F	CH_50	A	g	FTIR-A2	IV	0.5	0.1	Mid	0.60	0.42	Medium
F	CH_65	A	a	FTIR-A2	IV	0.2	0.0	Low	0.00	0.00	≤detection limit^f^
F	CH_66	A	a	FTIR-A2	IV	0.4	0.0	Mid	0.00	0.00	≤detection limit^f^
F	CH_69	A	b	FTIR-B1	IV	0.5	0.1	Mid	0.00	0.00	≤detection limit^f^
F	CH_72	A	a	FTIR-A1	IV	0.5	0.1	Mid	0.14	0.11	Low
F	CH_81	A	e	FTIR-A1	IV	0.7	0.1	Mid	0.01	0.02	Low
F	CH_95	F	f	FTIR-A2	V	0.2	0.0	Low	0.08	0.08	Low
F	CH_96	A	b	FTIR-B2	IV	0.4	0.0	Mid	1.14	0.16	High
F	CH_160	A	b	FTIR-A1	IV	0.7	0.0	Mid	0.00	0.00	≤detection limit^f^
F	P01_1	A	a	FTIR-A1	IV	0.7	0.0	Mid	0.11	0.01	Low
F	P03_1	A	a	FTIR-A1	IV	0.6	0.1	Mid	0.14	0.01	Low
O	2/27/S	A	a	FTIR-A1	IV	0.5	0.1	Mid	0.15	0.10	Low
O	6/27/S	A	a	FTIR-A1	IV	0.5	0.2	Mid	0.14	0.11	Low
O	1/29 AGES	A	b	FTIR-A1	IV	0.3	0.1	Low	0.00	0.00	≤detection limit^f^
O	3/22 AGES	A	b	FTIR-B2	IV	0.3	0.1	Low	0.00	0.00	≤detection limit^f^
O	CVUAS 2492	A	a	FTIR-A1	IV	0.4	0.0	Mid	0.12	0.09	Low
O	CVUAS 9660	A	a	FTIR-A1	IV	0.4	0.0	Mid	0.16	0.14	Low
O	CVUAS 9659	A	a	FTIR-A1	IV	0.4	0.0	Mid	0.30	0.25	Low

a *Isolates obtained from pesticides (P), foods (F), or in association with outbreaks (O)*.

b *Toxin profiles A–G correspond to the presence of the following combinations of toxin genes: A, nhe, hbl, cytK; B, nhe, cytK, ces; C, nhe, hbl; D, nhe, cytK; E, nhe, ces; F, nhe; G, cytK*.

c *All cytK positive isolates detected in this study harbored cytK-2*.

d *Singleton (S) in the cluster A*.

e *Values represent absolute values normalized using the absolute value of the highly toxic reference strain NVH 0075/95 included in the same run. Strains were classified as low level, mid level, or high level enterotoxin producers in adaptation of Jeßberger et al. (2015): low <0.4, mid = 0.4–0.8, high > 0.8*.

f *The limit of detection was determined using a SMase dilution series. The lowest amount of SMase that yielded a positive test result after 20 min was 0.444 mU, with one unit of SMase being defined as the amount of SMase that will hydrolyse 1 μmol of TNPAL-sphingomyelin per minute (at pH 7.4 and 37°C). SMase levels of ≤ 0.400 mU yielded a negative result under the same test conditions*.

### FTIR spectroscopic analysis

**Figure 2:**
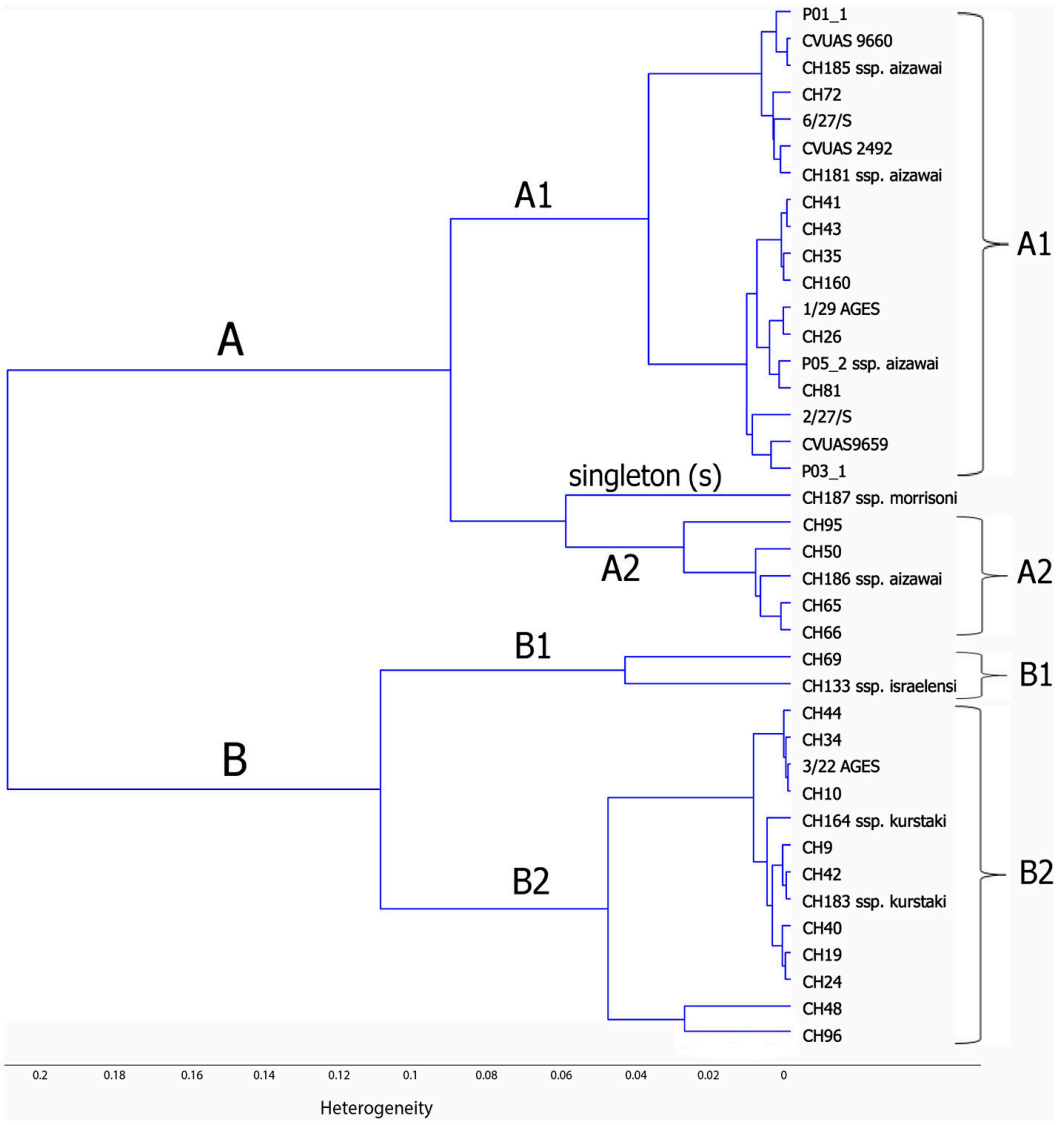
Dendrogram depicting similarity of FTIR spectra of *B. thuringiensis* isolated from different biopesticides, foods, and in connection to foodborne outbreaks. This analysis revealed two main clusters, designated FTIR-A and FTIR-B. Cluster FTIR-A comprised the *B. thuringiensis* ssp. aizawai strains. The biopesticide strain NB-176 (*B. thuringiensis* ssp. morrisoni) forms a singleton within this cluster. The two subclusters of FTIR-B can be assigned to *B. thuringiensis* ssp. *kurstaki* and *B. thuringiensis* ssp. israelensis, respectively.

Chemometric assisted FTIR spectroscopy was used to investigate the correlation between the FTIR spectra of *B. thuringiensis* bacterial strains isolated from different sources (biopesticides, food, and in association with foodborne outbreaks). This analysis revealed two main clusters, designated FTIR-A and FTIR-B (see Figure 2). Cluster FTIR-A comprised the *B. thuringiensis* ssp. *aizawai* strains. The biopesticide strain NB-176 (*B. thuringiensis* ssp. *morrisoni*) formed a singleton within this cluster. The two subclusters of the cluster FTIR-B can be assigned to *B. thuringiensis* ssp. *kurstaki* and *B. thuringiensis* ssp. *israelensis*, respectively. Although the biopesticide *B. thuringiensis* strains belonging to different serotypes clustered separately, they were intermixed with isolates from foods and foodborne outbreakes. For instance, the biopesticide *B. thuringiensis* ssp. *aizawai* strains were clearly separated from the serotypes *kurstaki* and *israelensis* but clustered together with isolates collected in the context of outbreak investigations (CVUAS2492, CVUAS9660, CVUAS9659, 2/27/S, 6/27/S) and isolates from foods (P01_1, P03_1) sprayed with *B. thuringiensis* ssp. *aizawai* directly before the harvest.

### Toxin gene profiling

As revealed by toxin gene profiling (Ehling-Schulz et al., 2006), all *B. thuringiensis* isolates in this study harbored one or more enterotoxin genes (see Table 4). The *nhe* gene was detected in all isolates. All biopesticide and outbreak isolates were also positive for *hbl* and, with the exception of biopesticide strain NB-176, also for *cytK*. Isolates were assigned to toxin profiles A (91%; *nhe, hbl, cytK*), C (3%; *nhe, hbl*), D (3%; *nhe, cytK*), and F (3%; *nhe*). As expected for *B. thuringiensis*, all *cytK* positive isolates exhibited the *cytK-2* variant of the cytotoxin K gene. The *ces* gene encoding the emetic toxin cereulide was not detected in any of the isolates.

### Cytotoxicity

**Figure 3:**
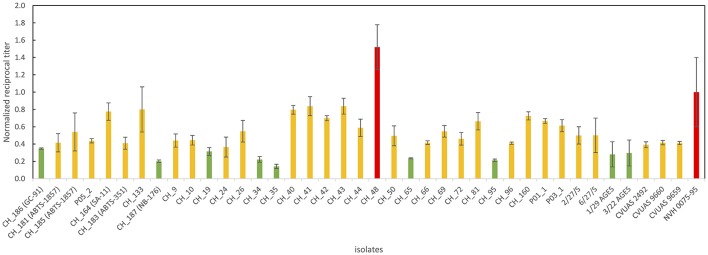
Overview of Vero cell cytotoxicity used to determine enterotoxin production. Values represent absolute values normalized using the absolute value of the highly toxic reference strain NVH 0075/95 included in the same run. The majority of the *B. thuringiensis* isolates including most biopesticide strains and the isolates linked to the outbreak in Germany in 2012 were classified as medium level enterotoxin producers (indicated in yellow). One food isolate was classified as high level enterotoxin producer surpassing the cytotoxicity of the highly toxic reference strain by a factor of 1.5 (indicated in red). Several isolates, including the biopesticide strains GC-91 and NB-176 were classified as low level enterotoxin producers (indicated in green).

All *B. thuringiensis* isolates included in this study showed cytotoxic effects in a Vero cell assay routinely used to assess the enterotoxicity of *B. cereus*. Although most isolates were classified as low or mid level enterotoxin producers, one isolate from rosemary (CH_48) exhibited cytotoxic effects 1.5x higher than the reference strain NVH 0075-95, known for its high-level enterotoxin production. An overview of all reciprocal titers determined in the Vero cell cytotoxicity assay is provided in Figure 3.

### SMase assay

**Figure 4:**
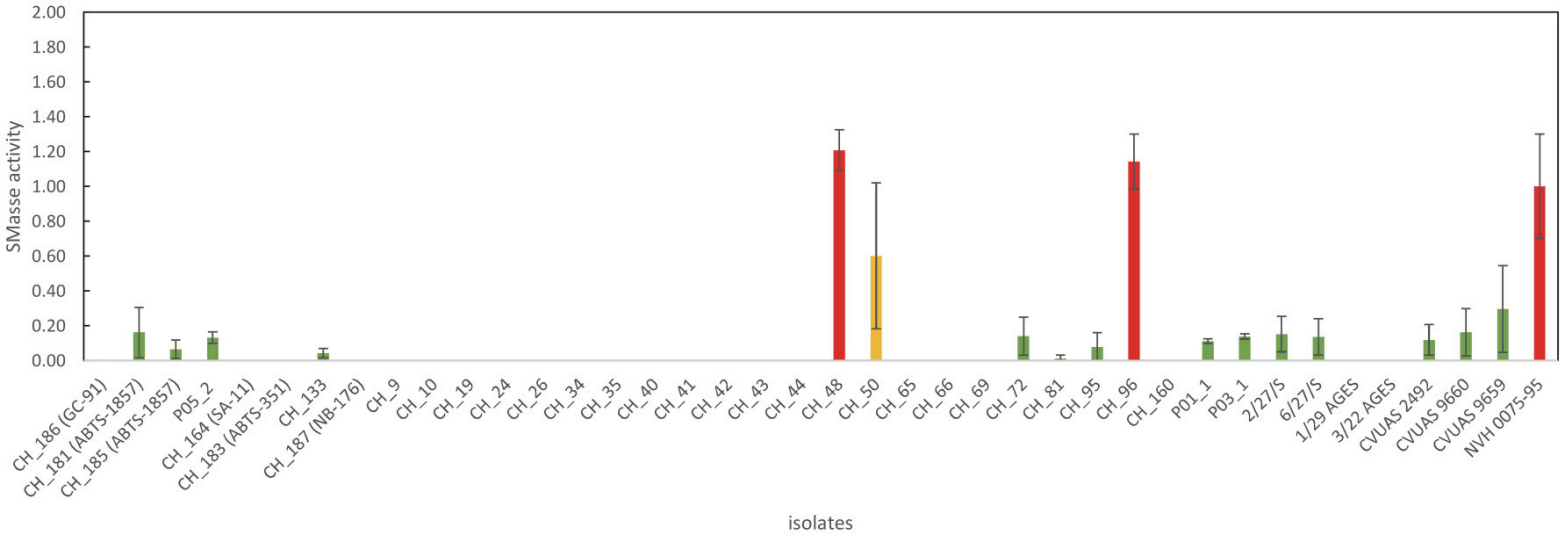
Overview of sphingomyelinase production of the *B. thuringiensis* isolates. Values represent Amplex Red results normalized using NVH 0075/95 (high-level SMase producer). For the majority of *B. thuringiensis* isolates, no SMase production was detectable (indicating that ≤ 0.400 mU of SMase were produced). Two isolates from food (CH_48 from rosemary and CH_96 from sushi) produced high levels of SMase. Interestingly, CH_48 also produced massive amounts of enterotoxins, surpassing the cytotoxicity of the highly toxic reference strain by a factor of 1.5.

An enzymatic assay was used to test all *B. thuringiensis* strains included in this study for their SMase activity. The reference strain NVH 0075-95, which is not only known for its high level of enterotoxin production but also for its high SMase activity (Doll et al., 2013), was included as a reference. All tested isolates, except three isolates from food (CH_48, CH_50, and CH_96), exhibited either no detectable SMase activity (detection limit: 0.004 U/mL) or produced low levels of SMase (Figure 4). *B. thuringiensis* ssp. *aizawai* strains ABTS-1857 and B401 as well as the *B. thuringiensis* ssp. *israelensis* isolate from Solbac showed low levels of SMase activity, while the other biopesticide strains tested negative.

## Discussion

Toxin gene profiling of the *B. thuringiensis* isolates obtained from biopesticides, foods, and outbreaks tested in this study revealed that the isolates commonly harbored enterotoxin genes. This is consistent with previous studies reporting high prevalences of enterotoxin genes in *B. thuringiensis* originating from pasteurized milk (Zhou et al., 2008), rice (Ankolekar et al., 2009; Kim et al., 2014), organic vegetables (Kim et al., 2017), food (Rosenquist et al., 2005; Ngamwongsatit et al., 2008), and soil (Ngamwongsatit et al., 2008). The collected evidence suggests that enterotoxin genes are common among *B. thuringiensis* independent of their source, and including biopesticide and food strains.

Several studies have confirmed that the *B. thuringiensis* strains from various sources express Nhe and/or Hbl using commercially available enterotoxin immunoassays (Abdel-Hameed and Landén, 1994; Damgaard, 1995; Hansen and Hendriksen, 2001; Rosenquist et al., 2005; Ankolekar et al., 2009; Kim et al., 2014). Damgaard (1995) screened various *B. thuringiensis* based biopesticides Bactimos, DiPel, Florbac FC, Foray 48B, MVP, Novodor FC, Turex, VecTobac, and XenTari for the presence of diarrheal enterotoxins using the *Bacillus* diarrhoeal enterotoxin visual immunoassay (BDE-VIA) kit provided by Tecra (Tecra diagnostics, Roseville, Australia). With the exception of one biopesticide, which lacks viable *B. thuringiensis* spores, all biopesticidal products yielded a positive result (Damgaard, 1995). Hansen and Hendriksen used the same assay to demonstrate Nhe expression in biopesticide strains HD-1 (Dipel ES) and HD-567 (Hansen and Hendriksen, 2001). Rosenquist et al. (2005) showed that strains contained in the biopesticides Dipel, Bactimos, and Vectobac all tested positive for both Nhe and Hbl expression using BDE-VIA (Tecra diagnostics) and the *B. cereus* enterotoxin reverse passive latex agglutination kit (BCET-RPLA, Oxoid, Basingstoke, UK), respectively. However, it was not further specified if Dipel ES (*B. thuringiensis* ssp. *israelensis*, strain HD-1) or Dipel DF (*B. thuringiensis* ssp. *kurstaki*, strain ABTS-351) was tested.

Cell culture assays have been reported to allow for more sensitive detection of *Bacillus* diarrheal enterotoxins than immunological assays (Buchanan and Schultz, 1994) and to enable classification of strains into low, mid, and high level enterotoxin producers (Jeßberger et al., 2015). Information on *B. thuringiensis* enterotoxicity based on cell culture assays is scarce and the few studies published so far (Damgaard et al., 1996; Gaviria Rivera et al., 2000) comprised only few biopesticide strains. Damgaard et al. (1996) tested food isolates from pasta (*n* = 5), pitta bread (*n* = 1) and milk (*n* = 1), as well as the three biopesticide strains HD-1, NB-125, and HD-567 representing *B. thuringiensis* serotypes *kurstaki, tenebrionis*, and *israelensis*, respectively. It could be demonstrated that all culture supernatants, except that from one strain isolated from milk, inhibited protein synthesis in a Vero cell assay. Our current study adds Vero cytotoxicity data for various other biopesticide strains including ABTS-1857 (serotype *aizawai*), which has been discussed as causative agent of a foodborne outbreak in Germany in 2012 (EFSA BIOHAZ Panel, 2016).

In recent years, it has become increasingly evident that specific host settings and parameters are playing a crucial role for enteropathogenicity of *B. cereus*. In particular, the role of spore survival, germination, and adhesion under conditions mimicking the host and the impact of intestinal conditions on enterotoxin synthesis have been investigated (Wijnands et al., 2009; Berthold-Pluta et al., 2015; Jeßberger et al., 2017). However, further studies will be needed to fully understand the relationship of enterotoxin formation, *in vitro* cytotoxicity, and the ability of a strain to cause clinical disease.

With one exception, all *B. thuringiensis* isolates tested in this study were assigned to *panC* type IV, including all biopesticide strains. As *B. thuringiensis* strains have previously been reported in association with *panC* types II, III, IV, V, and VI (Guinebretière et al., 2008, 2010; Carroll et al., 2017), the close genetic relatedness observed among the biopesticide strains and isolates collected from foods in our study foster the hypothesis that biopesticide strains can indeed be detected on foodstuff. Comparative genomics of the *B. thuringiensis* population showed that strains that belong to the so-called clade 2, which comprises strains of *panC* types IV and V (Ehling-Schulz and Messelhäusser, 2014), possesss highly potent insecticidal toxins and carry multiple *cry* genes (Zheng et al., 2017; Méric et al., 2018). Due to their high invertebrate toxicity, these *B. thuringiensis* strains are ideal candidates for biopesticides and strains commonly used as biopesticides belong to this phylogenomic group. Consistent with this hypothesis, *panC* IV strains frequently originate from natural environments (soil, water, air, plants), various foods, and from insects (Guinebretière et al., 2008). Nevertheless, *panC* IV strains can also be found associated with foodborne outbreaks of diarrheal disease (Guinebretière et al., 2008; Jeßberger et al., 2015; Glasset et al., 2016). Food poisoning risk however, has been suggested to be highest for *B. cereus* group isolates that belong to *panC* group III (Guinebretière et al., 2010), a group not detected among any of the isolates tested in this study. Growth temperature ranges vary between *panC* groups, with group IV being classified as mesophilic (10–45°C) and group V being classified as moderately psychrotolerant (8–40°C) (Guinebretière et al., 2008). *B. cereus* group strains assigned to *panC* IV have been shown to particularly frequently harbor *cytK* (Miller et al., 2018). Consistent with the findings in this study, Caco2 cytotoxicity of *panC* IV strains has been reported to vary greatly (Guinebretière et al., 2008; Jeßberger et al., 2015; Miller et al., 2018).

The relatedness of the isolates characterized in this study was further assessed using *panC-*based SplitsTree and FTIR spectroscopic analysis. The clusters obtained using these techniques, as well as the combined information derived from toxin gene profiling, *panC* typing, and cytotoxicity and SMase assays were used to determine characterization patterns. Several biopesticide strains exhibited characterization patterns that could not be distinguished from those originating from food or outbreak isolates (Table 5). The patterns obtained from *B. thuringiensis* isolated in the German outbreak in 2012 associated with lettuce previously treated with ABTS-1857 (XenTari) were identical to the one obtained from ABTS-1857. This is consistent with FTIR data generated by the authorities (EFSA BIOHAZ Panel, 2016). In addition, the *B. thuringiensis* from B401 used to control bee pests was indistinguishable from isolates obtained from self-surveillance food samples of a honey producer, and GC-91 (Agree) was indistinguishable from one food isolate. B. thuringiensis isolates were detected at a level of 3 × 104 cfu/g in the salad sample implicated in the outbreak in Germany in 2012 (EFSA BIOHAZ Panel, 2016) and at the same levels of 3 × 104 CfU/g in the honey samples from self surveillance (this study), emphasizing that *B. thuringiensis* used as biopesticide can enter the food production and be found in foods at retail level at high levels. In both cases the biopesticides were applicated directly before harvest.

**Table 5:** Overview of biopesticide isolates and identical characterization patterns (toxin gene profile, *panC* type, *panC-*based SplitsTree cluster, FTIR cluster, cytotoxicity in a Vero cell assay, and SMase activity) determined for food or outbreak isolates.

**Biopesticide strain^a^**	**Toxin profile^b^**	***panC* type**	**SplitsTree cluster**	**FTIR cluster**	**Enterotoxin production in Vero assay**	**SMase production**	***n* food isolates with identical pattern**	***n* outbreak isolates with identical pattern**
GC-91	A	IV	a	FTIR-A2	Mid	≤detection limit	1 (tarragon)	0
ABTS-1857 and B401^c^	A	IV	a	FTIR-A1	Mid	Low	3 (vegetable juice, 2 honey samples)	5 (3 salad samples Germany 2012; 2 human feces samples Austria 2013)
SA-11 and ABTS-351^d^	A	IV	b	FTIR-B2	Mid	≤detection limit	3 (spices)	0
Solbac	A	IV	c	FTIR-B1	Mid	≤detection limit	0	0
NB-176	C	IV	d	FTIR-S	Low	≤detection limit	0	0

*Nineteen isolates exhibited a pattern not identified in a biopesticide strain and were therefore not included in this table*.

a *In case no strain ID was provided on the product, trade names are used*.

b *Toxin profiles A–G correspond to the presence of the following combinations of toxin genes: A, nhe, hbl, cytK; B, nhe, cytK, ces; C, nhe, hbl; D, nhe, cytK; E, nhe, ces; F, nhe; G, cytK*.

c *Both ABTS-1857 and the B401 biopesticide isolates included in this study exhibited the same characterization pattern, which was detected again in isolates from foods and outbreaks*.

d *SA-11, ABTS-351, and three isolates from spices exhibited identical characterization patterns*.

*B. thuringiensis* based biopesticides were developed as a non-toxic alternative to chemical pesticides. They have been successfully applied in large-scale pest eradication for decades and allowed for a significant reduction of the use of chemical insecticides (Bravo et al., 2011). There are indications that enterotoxins are expressed by *B. thuringiensis* during septicemia in a target insect and therefore may contribute to the insecticidal effect of pesticides that contain not only crystal proteins, but also the organism itself (Kyei-Poki et al., 2007). Biopesticides exclusively relying on the insecticidal effects of purified *B. thuringiensis* crystals represent a safe alternative to formulations containing both crystals and viable endospores and have no known adverse effects on human health.

Our findings show that many *B. thuringiensis* biopesticide strains exhibit mid-level cytotoxicity in a Vero cell assay and that some of these strains cannot be differentiated from isolates obtained from foods or associated with outbreaks. Thus, we demonstrate that the use of *B. thuringiensis* strains as biopesticides may represent a food safety risk, underlining the importance of assessing the hazardous potential of each strain and formulation used. However, our findings also provide a novel explanation for the low number of clinical cases of diarrheal disease linked to *B. thuringiensis* over the last decades. With the exception of the low level enterotoxin producers GC-91 and NB-176, all other *B. thuringiensis* biopesticide strains tested exhibited mid level enterotoxicity. Nevertheless, compared to *B. cereus s.s*. their hazardous potential may be limited due to the lack of SMase, an important virulence factor complementing Nhe and Hbl induced cytotoxicity. We did not detect SMase production in the biopesticide strains GC-91, SA-11, ABTS-351, and NB-176. By contrast, biopesticide strains B401, ABTS-1857, and Solbac produced low levels of SMase. This is particularly interesting, as ABTS-1857 was implicated in the salad-related outbreak in Germany in 2012. Thus, further research should be focused on fully understanding the role of SMase in enteropathogenicity of *B. cereus s.l*. Such research will not only lead to a better understanding of the mechanisms of enteropathogenicity in *B. cereus*, but could also contribute to a better risk assessment of *B. thuringiensis* strains used as biopesticides.

## Conclusion

We demonstrated that most *B. thuringiensis—*including most biopesticide strains—tested in this study represent mid level enterotoxin producers. Several biopesticide strains could not be differentiated from isolates obtained from foods or associated with outbreaks based on *panC* type, SplitsTree and FTIR analysis, toxin gene profiles, cytotoxicity, and SMase production. Our data therefore suggests that biopesticide strains may be detected on foods after harvesting and that *B. thuringiensis* based biopesticides may pose a risk to consumer health. However, we also hypothesize that the hazardous potential of many commercially used *B. thuringiensis* strains might be limited due to low SMase production. The data presented in this study are a crucial contribution toward improved risk assessment of foodborne *B. thuringiensis*.

## Author contributions

SJ and ME-S conceived and designed the study. SJ, EK, NH, and RS carried out the experiments. PB, SG, RS, and MC contributed strains. MB performed the chemometric analysis of FTIR spectral data, SJ and ME-S analyzed and interpreted the data. SJ and ME-S wrote the manuscript. All authors revised and approved the final manuscript.

### Conflict of interest statement

The authors declare that the research was conducted in the absence of any commercial or financial relationships that could be construed as a potential conflict of interest.
